# Improving the Sensitivity of Task-Related Functional Magnetic Resonance Imaging Data Using Generalized Canonical Correlation Analysis

**DOI:** 10.3389/fnhum.2021.771668

**Published:** 2021-12-14

**Authors:** Emmanouela Kosteletou, Panagiotis G. Simos, Eleftherios Kavroulakis, Despina Antypa, Thomas G. Maris, Athanasios P. Liavas, Paris A. Karakasis, Efrosini Papadaki

**Affiliations:** ^1^Institute of Applied and Computational Mathematics, Foundation for Research and Technology – Hellas (FORTH), Heraklion, Greece; ^2^Computational Biomedicine Laboratory (CBML), Institute of Computer Science, Foundation for Research and Technology – Hellas (FORTH), Heraklion, Greece; ^3^Department of Psychiatry, School of Medicine, University of Crete, Heraklion, Greece; ^4^Department of Radiology, School of Medicine, University of Crete, Heraklion, Greece; ^5^Department of Medical Physics, School of Medicine, University of Crete, Heraklion, Greece; ^6^School of Electrical and Computer Engineering, Technical University of Crete, Chania, Greece; ^7^Department of Electrical and Computer Engineering, University of Virginia, Charlottesville, VA, United States

**Keywords:** task-related fMRI, signal sensitivity, fMRI, gCCA method, action observation, signal intensity

## Abstract

General Linear Modeling (GLM) is the most commonly used method for signal detection in Functional Magnetic Resonance Imaging (fMRI) experiments, despite its main limitation of not taking into consideration common spatial dependencies between voxels. Multivariate analysis methods, such as Generalized Canonical Correlation Analysis (gCCA), have been increasingly employed in fMRI data analysis, due to their ability to overcome this limitation. This study, evaluates the improvement of sensitivity of the GLM, by applying gCCA to fMRI data after standard preprocessing steps. Data from a block-design fMRI experiment was used, where 25 healthy volunteers completed two action observation tasks at 1.5T. Whole brain analysis results indicated that the application of gCCA resulted in significantly higher intensity of activation in several regions in both tasks and helped reveal activation in the primary somatosensory and ventral premotor area, theoretically known to become engaged during action observation. In subject-level ROI analyses, gCCA improved the signal to noise ratio in the averaged timeseries in each preselected ROI, and resulted in increased extent of activation, although peak intensity was considerably higher in just two of them. In conclusion, gCCA is a promising method for improving the sensitivity of conventional statistical modeling in task related fMRI experiments.

## Introduction

Functional Magnetic Resonance Imaging (fMRI) is one of the most popular methods for detecting systematic changes in regional brain activity during the performance of cognitive tasks. Brain activity is measured by detecting local changes of Blood Oxygenation Level Dependent (BOLD) signal which originates from systematic variations in blood oxygenation levels over time, typically between periods of active engagement in a given task and periods of rest or engagement in a comparison (reference or baseline) task.

Various statistical methods have been used to detect these signal variations, with General Linear Model (GLM) being one of the most popular. GLM statistical analysis is performed independently for each voxel and therefore is univariate. A univariate method, such as GLM, assumes neighboring voxels to be independent, without taking into consideration the spatial covariance structure of each voxel. An inherent limitation of GLM is that it does not take into account commonly occurring spatial dependencies between voxels (Zarahn et al., 1997). Spatial smoothing is typically applied to overcome this limitation which, in turn, may obscure smaller activity sources (Yang et al., 2018).

Multivariate analysis methods are being increasingly employed for fMRI data analysis, which can take into account multiple sources of variability simultaneously, including Independent Component Analysis (ICA; Calhoun et al., 2009), sparse Partial Least Squares (PLS) regression (Monteiro et al., 2015) and Canonical Correlation Analysis (CCA). The latter was first introduced by Hotelling (1936), and was designed to describe linear associations between two random variables so that their cross-correlation is maximized, and can be considered as a multivariate extension of the GLM (Thompson, 2005). Variants of CCA have been proposed to overcome crucial limitations of ordinary CCA, mainly poor specificity (Friman et al., 2001; Nandy and Cordes, 2004). One of these algorithms is generalized CCA (gCCA; Kettenring, 1971), a method applicable to more than two random vectors.

Both CCA and gCCA have been applied to fMRI data analysis. Friman et al. (2001) used CCA to successfully detect homogenous maps of the brain by combining subspace modeling of the expected task-related hemodynamic response and estimation of local spatial dependencies. Li et al. (2009) applied gCCA to multiple fMRI datasets in an attempt to separate distinct temporal sources within each dataset and to describe the correlation profiles of each source across different datasets. In contrast, Afshin-Pour et al. (2012) highlighted the possibility that several subjects may share an unknown spatial response (or spatial map) to a specific experimental manipulation, but may show different temporal responses. I n a previous report (Karakasis et al., 2020), a two-stage gCCA method was introduced for single-task multi-subject fMRI analysis, under the assumption of a common task-related set of spatial and temporal responses. The goal of this approach is to capture the basic features of the fMRI signal by taking into consideration both the common task-related spatial component and the common spatial components associated with ongoing, background activity. The proposed realization of gCCA computes the common task-related temporal component to derive an estimate of the associated common task-related spatial component and construct the respective activation map.

The goal of the present work was to explore the capacity of an unsupervised method for signal processing in improving the sensitivity of conventional statistical modeling of block-design task-related fMRI data. This approach relies on gCCA applied to the data after standard preprocessing steps (co-registration to MNI space, anatomic normalization, band-pass filtering/detrending, motion correction, and spatial smoothing). To demonstrate the potential utility of the gCCA algorithm we applied it to data from a previous fMRI experiment of action observation (Savaki et al., 2021). Specifically, we used data from two tasks identical in all design and stimulus characteristics with the exception of the precise kinematics of the observed action. Reference blocks involved observation of a static snapshot from the action clip presented during the “active” blocks, to further reduce visual differences between active and reference blocks (no decision or response was required from participants throughout the task). Initially, whole-brain activation maps were obtained by applying the standard GL model to (i) fMRI data submitted to conventional preprocessing and (ii) conventional preprocessing followed by gCCA. To characterize the effect of gCCA in more detail, we also extracted data on the extent and degree of estimated activation from first-level (person-specific) T-maps focusing on four key regions of the brain network known to be involved in action observation (Simos et al., 2017; Savaki et al., 2021). These regions were selected based on three additional criteria: (1) they cover both posterior (occipitotemporal and postcentral gyrus) and anterior sections of the brain (dorsolateral prefrontal), (2) they include both sensory processing (extrastriate visual and primary somatosensory) and motor representation cortex (dorsal and ventral premotor) areas, and (3) they display a wide range of signal intensities based on our earlier experiment on action observation (strong signal in occipitotemporal and dorsal premotor, and weaker, more variable signal across participants in ventral premotor and primary somatosensory cortex).

## Materials and Methods

### Participants

Functional Magnetic Resonance Imaging data were obtained from 25 healthy adults (mean age = 28.4, SD = 4.3 years; 11 men) without history of neurological or psychiatric disorder, sensory or motor deficit (convenience sample recruited through ads posted in the Voutes campus of the University of Crete). They all had normal or corrected to normal vision and provided written consent in accordance to the declaration of Helsinki. The study was approved by the Ethics Committees of (i) the University Hospital of Crete and (ii) the Foundation for Research and Technology – Hellas (FORTH).

### Stimuli and Tasks

Participants completed two passive, action observation tasks, which were identical in all respects with the exception of the specific kinematic characteristics of the observed action. Each task comprised four “active” 35 s blocks, alternating with four 35 s baseline blocks. A video clip illustrating a two-movement action sequence was presented 6 times within each “active” block. The stimulus set-up was identical across blocks and conditions, presenting a female person sitting behind a table. A white tea cup was positioned on the table and a ceramic bowl 30 cm in diameter was located on a smaller table right next to the person’s head. The “Fast to cup – Slow to person” condition (fs-P) consisted of a rapid grasping movement toward the tea cup (time duration equal to 1,400 ms and average velocity equal to 0:36 m/sec), followed by a much slower movement that brings the cup to the person’s mouth (time duration equal to 4,033 ms and average velocity equal to 0:12 m/sec). The “Slow to cup – Fast to person” condition (sf-P) consisted of a slow grasping movement toward the tea cup (time duration equal to 4,033 ms and average velocity equal to 0:12 m/sec), followed by a much faster movement that brings the cup to the person’s mouth (time duration equal to 1,400 ms and average velocity equal to 0:36 m/sec). Clip duration was always 5,433 ms, with a 400 ms blank screen between successive repetitions of each clip. The stimulus in the static baseline blocks consisted of the first frame of the corresponding video clip, which was repeated six times (duration = 5,433 ms) interspersed with a 400 ms blank screen. In both experiments a white cross for fixation remained on the screen for the entire duration of the recording, with the exception of a 3 s period between blocks during which a blank screen was presented to allow participants to move their eyes freely and blink if needed. Participants were asked to keep fixating the cross when appeared on the screen, and keep their hands resting on their stomach. Additional details on the stimuli and task can be found in Savaki et al. (2021).

### Image Acquisition and Conventional Preprocessing

Functional Magnetic Resonance Imaging data were acquired using an upgraded 1.5T Siemens Vision/Sonata scanner (Erlangen, Germany) with powerful gradients (Gradient strength: 45 mT/m, Gradient slew rate: 200 mT/m/ms) and a standard four channel head array coil. For the BOLD-fMRI, a T2*-weighted, fat saturated 2D-FID-EPI sequence was used with the following parameters: repetition time (TR) 3,500 ms, echo time (TE) 50 ms, field of view (FOV) 192 × 192 × 108 mm (x; y; z), acquisition voxel size 3 × 3 × 3 mm. Whole brain scans consisted of 36 transverse slices with 3.0- mm slice thickness and no interslice gap. The timeseries recorded in each condition comprised 80 volumes (time points), with 40 volumes recorded during observation of repeated person-directed action and 40 volumes recorded during observation of a static hand. The first 5 volumes of each time-series were ignored in the analyses as is customary in fMRI studies.

Additionally, high resolution anatomical images were acquired sagittally, using a 3D magnetization-prepared rapid acquisition gradient echo sequence (3D-MPRAGE) with the following parameters: TR 9.8 ms, TE 4.6 ms, flip angle 8 deg, inversion time (TI) 922 ms, FOV 180 × 230 (x; z), with acquisition voxel size of 0.98 × 0.98 (x; z) and slice thickness of 1 mm.

Image preprocessing was performed in SPM12. Initially, EPI scans were spatially realigned to the first image of the first time-series using second-degree B-spline interpolation algorithms and motion-corrected through rigid body transformations (three translations and three rotations about each axis). Next, images were spatially normalized to a common brain space (MNI template), smoothed using an isotropic Gaussian filter (FWHM = 8 mm), and high pass filtered with a time constant of 128 s.

### Additional Preprocessing Using Generalized Canonical Correlation Analysis

In a previous report of ours (Karakasis et al., 2020), a generative model was proposed which takes into account the common task-related spatial and temporal responses, as well as the common spatial responses that are related to the background hemodynamic activity attributed to resting state networks. Specifically, under the setting of a task-related multi-subject experiment, the aforementioned model assumes that the recordings of each subject can be expressed as a linear superposition of three groups of components. The first group comprises the spatial and temporal responses to the experimental stimuli and are assumed to be common across different subjects up to possibly different intensities. The second group expresses the spatial and temporal responses that are attributed to the existence of resting state networks. The model assumes that these responses have common, across subjects, manifestations in space, under the assumption of common spatially organized resting state networks, while the temporal responses of these networks may vary across subjects. Finally, the third group captures the uncommon responses of each participant, which do not belong to the first two groups and can be considered as (strong) additive noise, independent across participants.

In earlier work gCCA had been successfully applied in estimating a linear subspace which is “common” to a collection of sets of random variables (Ibrahim and Sidiropoulos, 2019) and latter applied to estimate a basis of the subspace that is spanned by the common spatial responses (task-related or not; Karakasis et al., 2020). Projecting the fMRI data of all participants onto this subspace helps to suppress any spatial responses that are not shared by all participants and, as a result, provides a noise reduction effect, which could potentially increase the SNR in both weakly and strongly activated regions. In our previous work and in the present analyses the gCCA algorithm is supplemented by a data-driven criterion for estimating the proper dimension for modeling the common subspace.

### Statistical Analyses

The two fMRI datasets (conventionally preprocessed designated as “original” and conventionally preprocessed followed by gCCA [“gCCA-processed”] were submitted to a fixed effects General Linear Model (Friston et al., 1995) separately for each participant and task. The model included two condition regressors of interest (active, static). Contrasts of interest (fs-P or sf-*p* > the corresponding static baseline) were thresholded at *p* < 0.001 uncorrected.

In whole-brain analyses, individual contrast *t*-value images were input to the second level random effects analysis to estimate the generalizability of activations. The main analysis involved one-sample *t*-tests (s-f to person vs. static baseline and f-s to person vs. static baseline). Supplementary analyses involved pairwise comparisons of resulting contrast maps between original and gCCA-processed data, separately for each task. Second level activations were assessed for statistical significance by applying a threshold of *p* < 0.05 with family wise error (FWE) correction for multiple comparisons at the cluster level, with an initial voxel-wise threshold of *p* = 0.001. Monte Carlo simulation (Slotnick, 2017) was applied when necessary (in cases where voxel clusters were smaller than the minimum number required to obtain FWE-adjusted significance of *p* < 0.05) using the estimated smoothness of our functional data (10 mm). This resulted in a minimal cluster size of 58 voxels at a threshold of *p* < 0.001 uncorrected to achieve correction for multiple comparisons for *p* < 0.05. Anatomic identification of active clusters was performed using the SPM Anatomy Toolbox (v2.2; Eickhoff et al., 2004).

In region-specific analyses, we computed the extent of activation (number of voxels exceeding a fixed criterion of *p* < 0.01 uncorrected) and peak activation (*T* value) in four ROIs where significant, activation clusters were found and which are known to serve as key regions of the brain network involved in action observation (Savaki and Raos, 2019). These masks were manually drawn using the WFU PickAtlas toolbox, taking care to constrain them within the anatomical borders of the following regions in the Automated Anatomical labeling atlas (Tzourio-Mazoyer et al., 2002): (1) Extrastriate Body Area (EBA; comprised of Brodmann’s area [BA] 37), (2) primary somatosensory area (SI, BA 2/3), (3) dorsal premotor area (PMd, BA 6), and (4) ventral premotor area (PMv; BA 6). Areas EBA, PMd, and PMv were drawn in the right hemisphere and area SI in the left hemisphere where the effect of action observation on activation was greatest in our previous work (Simos et al., 2017; Savaki et al., 2021). The effect of the additional processing through gCCA on the degree and/or extent of activation in each of the four ROIs was assessed through repeated measures ANOVAs with task (sf-P, fs-P), ROI (EBA, SI, PMd, PMv), and data set (original vs. gCCA-processed) as within-subjects variables.

Finally, the effect of the gCCA algorithm on the specificity of activations was estimated by computing the extent of active voxels (at *p* < 0.01 uncorrected in first-level maps) and peak activation (*T* values) within a whole-brain, inclusive mask of CSF and white matter. Additionally, SNR was estimated for the original and gCCA-processed timeseries, averaged across all voxels within the aforementioned ROIs. SNR was estimated as the ratio of the mean signal of the fMRI timeseries across all voxels within a given ROI divided by the standard deviation of the average timeseries across all voxels within a large manually drawn region encompassing periventricular white matter bilaterally (same across participants, using the WFU PickAtlas tool). In this manner the estimated noise signal reflected both equipment and physiological noise.

## Results

### Whole-Brain, Group-Level Analyses

**FIGURE 1:**
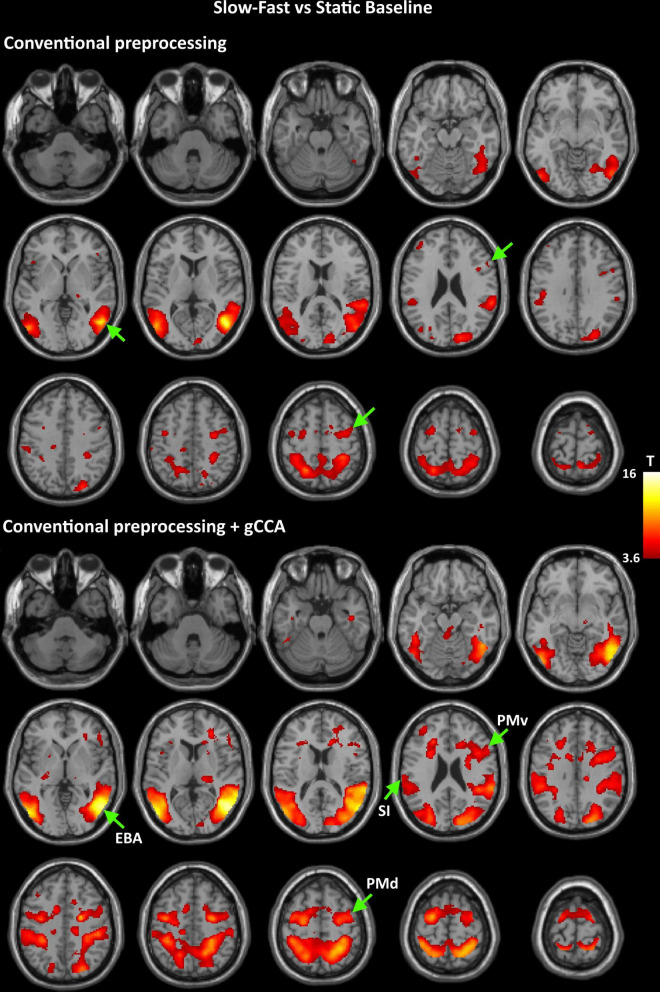
Group-level T maps of activation during observation of a slow to cup-fast to person action compared to the static reference blocks. Upper panel: data submitted to conventional preprocessing only; lower panel: data submitted to conventional preprocessing followed by gCCA. Arrows point to the four ROIs selected for region-specific analyses at the subject level (EBA: Extrastriate Body Area, PMd: dorsal Premotor area, PMv: ventral Premotor area, SI: primary somatosensory area).

**FIGURE 2:**
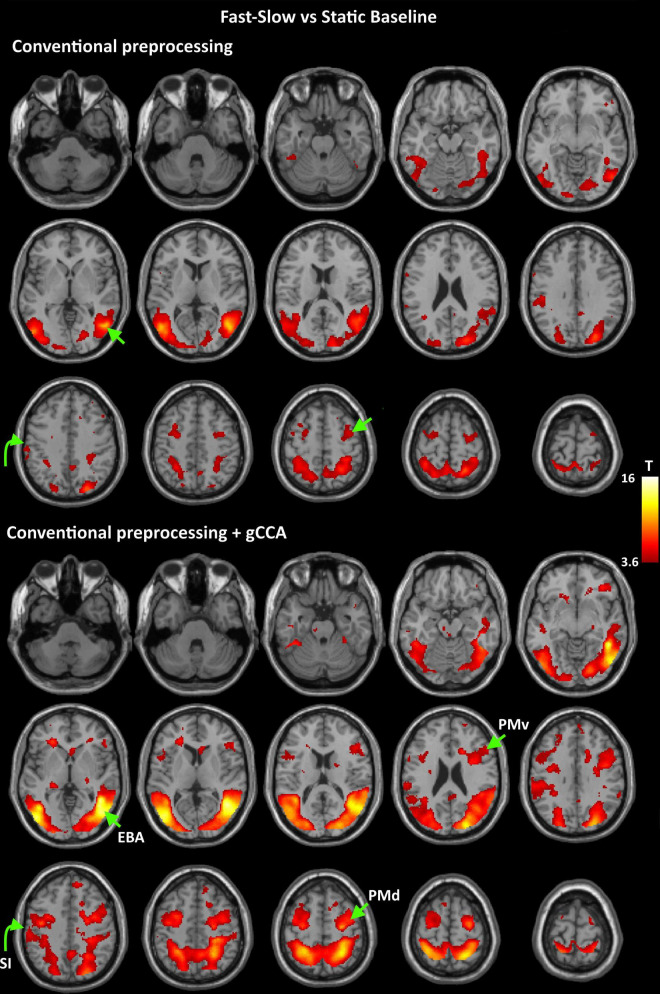
Group-level T maps of activation during observation of a fast to cup-slow to person action compared to the static reference blocks. Upper panel: data submitted to conventional preprocessing only; lower panel: data submitted to conventional preprocessing followed by gCCA. Arrows point to the four ROIs selected for region-specific analyses at the subject level (EBA: Extrastriate Body Area, PMd: dorsal Premotor area, PMv: ventral Premotor area, SI: primary somatosensory area).

Coordinates of significant activity clusters found in second-level whole-brain analyses (positive contrast at *p* < 0.05 corrected) and corresponding *T* values for original and gCCA-processed data are shown in Tables 1, 2 (Group-level T maps for original and gCCA-processed data are shown in Figures 1, 2). Inspection of group-level *T* values indicates that gCCA-processed data produced either similar (in 4 regions in the sf-P and in 5 regions in the fs-P task) or considerably higher degree of activation in several regions (in 11 regions in the sf-P and in 9 regions in the fs-P task). Significant activation clusters were found only in the gCCA-processed data in 7 and 6 regions, respectively. Whole-brain paired *t*-tests confirmed that the task vs. static baseline contrast values were significantly higher in the gCCA-processed as compared to the original data in 10 and 9 regions for the sf-P and fs-P tasks, respectively. It should be noted that gCCA-processed < original comparisons did not reach significance, although the corresponding one-sample *T* values were slightly different, in the following areas: SMA bilaterally (sF-P task), right EBA, right IPLv, and left SI (Fs-P task).

**TABLE 1:** MNI coordinates and *T* values of significant activations in whole-brain second-level analyses during observation of Slow-fast to Person action comparing original vs. gCCA-processed data.

			Original				gCCA-processed				Original < gCCA-processed
Brain area	BA	H	x	y	z	*T*	x	y	z	*T*	
EBA/MT	37	L	−50	−72	6	10.98	−48	−72	6	12.50	9.11
EBA/MT	37	R	46	−64	8	11.72	50	−62	6	14.63	7.78
ITG/FG	37	L	−42	−80	−6	7.30	−46	−74	−6	9.90	4.47
ITG/FG	37	R	70	−70	−6	7.70	50	−66	−10	10.47	4.04
SPL	7	L	−18	−64	56	4.86	−20	−58	60	9.56	7.06
SPL	7	R	28	−58	60	6.29	20	−58	60	10.40	–
IPLd	40	L	−26	−40	54	6.33	−36	−42	60	8.88	5.58
IPLd	40	R	32	−40	56	7.12	28	−48	58	10.80	6.29
IPLv	40	L	−52	−38	28	4.90	−50	−36	30	6.60	3.50
TPj	40	R	61	−38	18	8.22	62	−40	20	10.61	–
SI	3	L	–	–	–	–	−50	−26	32	5.40	
SI	3	R	–	–	–	–	46	−28	38	8.30	3.80
PMd	6	L	−26	−6	64	4.31^a^	−22	−4	62	9.70	–
PMd	6	R	40	−4	58	6.58	36	−4	58	8.10	4.48
PMv	6	L	–	–	–	–	−46	−2	42	5.10	–
PMv	6	R	42	8	24	5.15^a^	42	6	30	6.58	–
SMA	6	L	0	−2	58	4.67	−6	0	60	4.55	–
SMA	6	R	14	2	60	6.02	6	4	66	4.24	–
IFG	47	R	–	–	–	–	52	32	2	4.60	–
IFG	45	R	–	–	–	–	54	18	24	5.30	–
MFG	9/10	L	–	–	–	–	−34	44	24	4.68	–

**TABLE 2:** MNI coordinates and *T* values of significant activations in whole-brain second-level analyses during observation of Fast-Slow to Person action comparing original vs. gCCA-processed data.

			Original				gCCA-processed				Original < gCCA-processed
Brain area	BA	H	x	y	z	*T*	x	y	z	*T*	*T*
EBA	37	L	−50	−72	4	9.14	−46	−74	0	13.33	7.66
EBA	37	R	50	−66	4	14.66	50	−66	4	13.34	–
ITG/FG	37	L	−50	−70	−12	6.51	−46	−74	−8	10.17	4.40
ITG/FG	37	R	50	−68	−12	7.62	50	−64	−10	10.90	4.67
SPL	7	L	−32	−50	56	6.67	−18	−56	60	9.56	5.37
SPL	7	R	22	−54	60	7.24	24	−52	60	11.98	5.86
IPLd	40	L	−32	−48	56	7.24	−26	−44	56	9.50	5.56
IPLd	40	R	34	−50	58	6.49	32	−48	56	11.13	5.78
IPLv	40	L	−54	−30	34	6.41	−58	−30	30	5.55	–
TPj	40	R	60	−38	18	6.86	60	−44	18	9.22	–
SI	3	L	−48	−26	36	7.66	−54	−24	38	5.95	–
SI	3	R	–	–	–	–	32	−30	46	9.60	4.32
PMd	6	L	−34	−8	52	6.82	−26	2	48	7.00	–
PMd	6	R	36	−4	48	6.72	30	−12	58	10.30	5.08
PMv	6	L	−60	6	28	6.35^a^	−40	−8	38	7.50	–
PMv	6	R	–	–	–	–	40	0	36	6.68	–
SMA	6	L	–	–	–	–	–	–	–	–	–
SMA	6	R	–	–	–	–	–	–	–	–	–
IFG	47	R	–	–	–	–	40	40	−10	5.31	–
IFG	45	R	–	–	–	–	46	26	14	5.32	–
MFG	9/10	L	–	–	–	–	−50	10	30	3.60	–
MFG	9/10	R	–	–	–	–	46	10	34	5.37	–

*Abbreviations for Tables 1 and 2; EBA, extrastriate body area; ITG, inferior temporal gyrus; FG, fusiform gyrus; SPL, superior parietal lobule; IPL, inferior parietal lobule; d, dorsal; v, ventral; TPj, temporoparietal junction; SI, primary somatosensory area; PM, premotor cortex; SMA, supplementary motor area; IFG; inferior frontal gyrus; MFG, middle frontal gyrus; BA, Brodmann’s area; H, hemisphere; L, left; R, right; FEW p < 0.05 corrected. ^a^Significant activation after Monte Carlo simulation (threshold > 58 voxels). No voxels exceeded p > 0.05 uncorrected, for original > gCCA-processed. Significant voxel clusters in primary and secondary visual cortices are not shown.*

Negative contrasts were also computed in each dataset (i.e., deactivations indicated by sf-P or fs-*p* < static baseline differences). One-sample T maps featured sparse deactivations in both tasks and datasets, which did not show systematic anatomic distributions. Moreover, paired sample contrasts did not reveal significant effects of data set (gCCA-processed vs. original) for either task, even at a more liberal threshold of *p* < 0.001 uncorrected with a smaller extent threshold of 20 voxels.

Significant activity clusters in WM, and primarily in CSF, were found in 11/25 participants in the gCCA-processed data (mean_voxels_ = 16.7, SD_voxels_ = 32.9, range = 0–149, mean_T_ = 1.6; SD_T_ = 1.9, range = 0–5.1) but only in one participant in the original data (22 voxels, *T* = 3.6). However, this notable increase in active extracortical voxels was not associated with a corresponding increase of SNR in key cortical ROIs (*r* < 0.1).

### Single-Task, Individual Activation Maps

**FIGURE 3:**
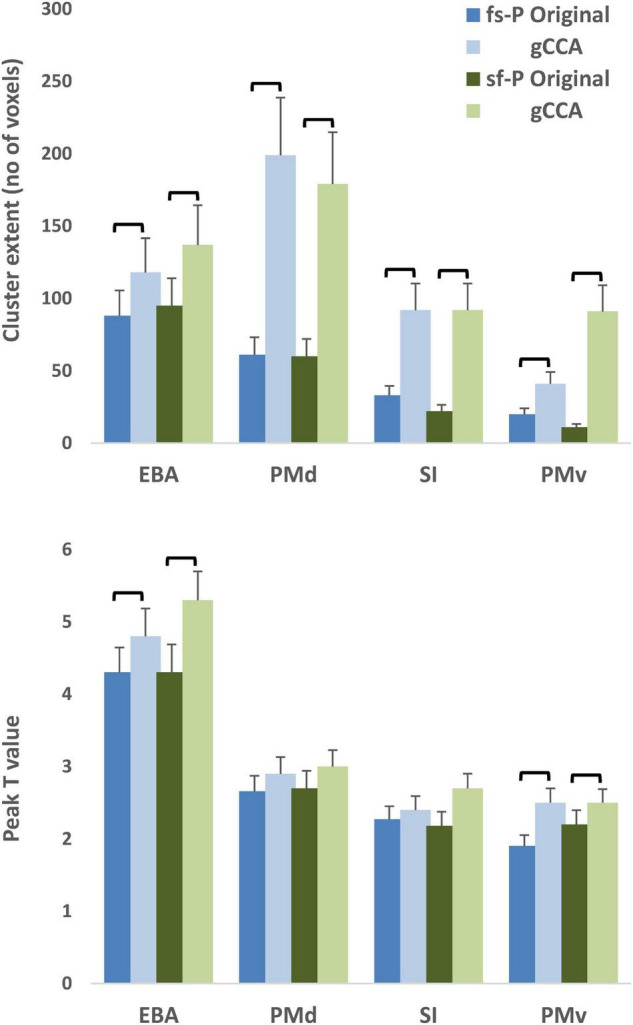
Average extent of activation (upper panel) and peak *T* values (lower panel) of activity clusters in four selected ROIs (Extrastriate body area [EBA], dorsal premotor area [PMd], ventral premotor area [PMv] in the right hemisphere, and left primary somatosensory area [SI]). Shades of blue represent data from the original dataset and green shades gCCA-processed data. Brackets indicate significant differences at *p* < 0.001 (upper panel, main effect of dataset) or *p* < 0.0125 (lower panel, Bonferroni-adjusted simple main effects). Vertical bars represent standard error. fs-P: Fast-Slow to Person, sf-P: Slow-Fast to Person.

Significantly greater extent of activation was noted in each of the four preselected ROIs as indicated by a main effect of dataset (original, gCCA-processed), *F*(1,24) = 12.69, *p* = 0.002 (see Figure 3). The effect of dataset on peak intensity varied across ROIs as indicated by a significant dataset by ROI interaction, *F*(3,72) = 20.62, *p* < 0.001. Simple main effect tests (evaluated at Bonferroni-adjusted *p* < 0.0125) revealed higher *T* values in the gCCA-processed dataset in EBA, *F*(1,24) = 33.83, *p* < 0.001, and PMv, *F*(1,24) = 8.45, *p* = 0.008. On average, *T* values increased by 28.1% (SD = 5.1%) in PMv, 8.9% (SD = 2.2%) in EBA, and 12.4% (SD = 1.9%) in SI, and remained unchanged in PMd (mean = −0.2%, SD = 13.1%).

Moreover, the likelihood of obtaining significant clusters in a given ROI was slightly higher in the gCCA-processed data as compared to the original data in PMv (20/25 [both tasks] vs. 15/25 in the fs-P and 17/25 participants in the sf-P, respectively) and SI (in the sf-P task only: 18/25 vs. 22/25 participants, respectively). In the remaining ROIs the frequency of significant clusters was very similar between datasets. On average, cluster extent increased by 381.3% (SD = 59.0%) in PMd, 409.3% (SD = 62.5%) in PMv, 614.5% (SD = 89.4%) in EBA, and 547.5% (SD = 77.6%) in SI.

These results were accompanied by an average increase of 62% in SNR in each of the four ROIs (across tasks). Specifically, average SNR in PMd was 168.7 (SD = 52.2) in the gCCA-processed and 106.1 (SD = 31.7) in the original data. Corresponding values in PMv were: 170.2 (SD = 52.8) and 106.4 (SD = 29.2); in EBA: 180.3 (SD = 58.3) and 112.8 (SD = 32.8); and in SI: 166.7 (SD = 51.3) and 104.3 (SD = 28.7). However, individual differences in SNR improvement following gCCA did not correlate significantly with percent change in either peak *T* value or extent of activation (*r* < 0.2).

## Discussion

In this study we explored the potential utility of an unsupervised multivariate method in improving the sensitivity of the conventional GLM to detect systematic BOLD changes in block-design fMRI experiments. Specifically, we used fMRI data from two tasks involving action observation known to activate a complex network of distributed brain regions in both hemispheres. The selected tasks were virtually identical in timing parameters and response requirements: both involving passive observation of a motor act executed by a third person. In this manner, background, resting brain activity was reinforced, given that gCCA models components related to continuous, resting-state as well as task-related BOLD responses. Conversely, reference blocks involved (passive) observation of a visual stimulus (common to both tasks) depicting the initial frame of the action presented in the “active” blocks. In this manner, perceptual and cognitive differences between reference and “active” blocks were kept at a minimum to ensure that activated regions would be largely restricted to those involved in action observation *per se*. While some of these regions (such as EBA, SPL, PMd) are known to display very robust activation during action observation, other purportedly crucial regions of the network (such as SI, PMv, and IFG) typically display weak and variable activation across participants. This expectation was corroborated by our second level, whole-brain results as indicated relatively small one-sample *T*-test values (Tables 1, 2). In sum, the present study was designed to explore the limits of gCCA to facilitate detection of weak task-related activity while, in conjunction with conventional GLM, maintaining the activations associated with resting neuronal activity at a minimum.

Whole-brain analyses support the specificity of gCCA-derived GLM results given that we did not detect activity clusters outside the broad network of regions known to be involved in action observation. The anatomic overlap between the two analyses is restricted to regions comprising this network. Importantly, the overlapping regions shown in Tables 1, 2 are virtually identical across tasks, further supporting the reliability of the proposed method. Extrastriate visual cortices are known to be involved in visual processing of both static and moving body parts (EBA; Filimon et al., 2007; Gazzola and Keysers, 2009); the primary somatosensory (hand area in the postcentral gyrus) may be involved in mapping effector-related properties of an observed action (Keysers et al., 2004) and related predictions; cortex in the superior parietal lobule may contribute to estimations related to visuomotor integration (Barany et al., 2014); inferior parietal regions could be involved in gesture representation (Sirigu et al., 1999) whereas adjacent TPj in body knowledge (Van Overwalle and Baetens, 2009); activity clusters were found in the dorsolateral (PMd) and mediodorsal premotor cortices (SMA), which are also crucial components of the sensorimotor system responsible for overt action execution, in agreement with previous meta-analyses (Caspers et al., 2010; Molenberghs et al., 2012). Finally, activity clusters were found in the ventral premotor and adjacent inferior and middle frontal gyri, which are potentially involved in specifying action intension (Becchio et al., 2008). In general, the anatomy of jointly activated areas across tasks observed in the present study is in agreement with the notion of a common network underlying the recognition of transitive, intransitive, and meaningless gestures independent of their type in humans (Villarreal et al., 2008) and non-human primates (Savaki, 2010; Savaki and Raos, 2019). Additional active voxels were detected in 11/25 participants but they were located in non-cerebral regions (subcortical white matter and, primarily, in ventricles), rather than in brain regions typically linked to known resting-state functional networks (e.g., Yeo et al., 2011). This spurious activity was likely represented by one or more of the common spatial subspace dimensions estimated through gCCA, which survived the purely data-driven method used to estimate the optimal number of spatial dimensions to retain in the gCCA-processed data. Thus, in future studies using the gCCA algorithm it is advisable to ensure that any spatial components showing significant (e.g., >50%) overlap with a CSF mask are excluded prior to reconstructing the voxel time-series.

The present results further support the added value of gCCA in improving the sensitivity of GLM. In whole-brain analyses this claim is supported by three sets of findings: Firstly, significant clusters of activity were detected, following gCCA, in two additional regions which have been proposed by previous studies and theoretical accounts to become engaged during action observation: the hand representation in the right primary somatosensory cortex and the right inferior frontal gyrus (BA 45). Secondly, direct comparisons between original (obtained through conventional preprocessing) and gCCA-processed data revealed significantly higher intensity of activation in the latter in several regions of the action observation network, 8 of which were common to both tasks (left EBA and SPL, right SI and PMd, bilateral ITG/FG and IPLd). The opposite trend (original > gCCA) was not observed in any region. Thirdly, direct comparisons of the two datasets on activation parameters in four representative ROIs revealed that the gCCA-processed data provided more robust activity clusters in less strongly activated regions (namely the right PMv and left SI) as indicated by greater extent of activation at the single-subject level. Interestingly, increased extent of activation of subject-specific clusters in the left SI was accompanied by significant enhancement of peak activation in the group-level analyses, although this was not the case for the right PMv. This failure probably illustrates the limits of the current implementation of gCCA in cases of activity clusters that are very weak and/or anatomically variable across participants.

A final note is in place regarding the effect of gCCA on peak cluster activations at the subject level. The current ROI-based analyses indicate that the contribution of gCCA on peak activation intensity was less pronounced than its effect on the corresponding extent of activation, as it was documented only for EBA and PMv clusters. Taken together our findings suggest that gCCA may serve as a useful step in fMRI data preprocessing, especially in less salient activation conditions, in order to identify and measure cluster activation parameters in certain areas of interest. The proposed model takes into account the common task-related spatial and temporal responses, as well as the common spatial responses that are related to the resting state networks. Within the gCCA framework, one can estimate a basis that spans the subspace of all common spatial responses (task-related or not). Moreover, this step can help suppress all the non-common spatial responses which are not represented by the derived components. In effect gCCA serves as a noise reduction tool that increases SNR in weakly (as well as in strongly) activated regions. The substantial improvement in SNR of the BOLD signal recorded in key brain regions in the present study is consistent with the notion that application of gCCA can help to effectively reduce the non-common sources of both physiological and instrument noise [as estimated by the temporal variability of the recorded signal in extracortical regions (cerebral white matter)]. It should be noted, however, that the behavior of gCCA in handling specific sources of noise, such as head motion, remains to be demonstrated in future analyses. Finally, the decision to apply gCCA should be weighted against the necessary compromise between sensitivity and specificity, which largely depends upon the nature of the specific research questions and experimental task(s) employed in a given study (Lieberman and Cunningham, 2009). The task of the present experiment was designed to reveal activations associated with cognitive processes, therefore subtler in comparison to sensorimotor activations; thus, high sensitivity was more preferable compared to specificity. Moreover, the anatomic layout of cerebral activations was largely known based on extensive prior work, so that potentially spurious cortical activations could be identified. Conversely, in tasks where high specificity is desirable and the anatomic layout of activations is not very well defined from prior work, the use of conventional GLM preprocessing might be a more appropriate, yet conservative approach to ensure optimal reliability of the results.

In conclusion, the present experimental results present a promising method for improving the sensitivity of the GLM to detect significant activity clusters in statistical method analysis, with only modest reduction in specificity (activations in areas outside the purported brain network involved in action observation). The results presented here constitute a basis for future studies in order to assess the impact of the proposed method in other experimental contexts, including various event-related designs. Application of gCCA to data derived from tasks that require a motor response and, therefore, are expected to produce much more robust sensorimotor neuronal activity, is also forthcoming to examine whether this method has a similar impact on SNR and task-related activation in these brain regions.

## Data Availability Statement

The data analyzed in this study is subject to the following licenses/restrictions: Raw structural MRI images may not become available to other users as they contain identifying information for individual participants. Requests to access these datasets should be directed to EP, fpapada@otenet.gr.

## Ethics Statement

The studies involving human participants were reviewed and approved by the University of Crete Hospital Ethics Committee and the Ethics Committee of the Foundation for Research and Technology – Hellas (FORTH). The patients/participants provided their written informed consent to participate in this study.

## Author Contributions

EKo collected image data, performed image and statistical analyses, and contributed to the writing of the manuscript. EP and TM performed and interpreted the MRI exams, edited, and critically reviewed the manuscript. DA and EKa collected image data, performed image analysis, and critically reviewed the manuscript. PS contributed to the design of the study, statistical analyses, and writing of the manuscript. AL and PK developed and implemented the gCCA algorithms, edited, and critically reviewed discussion manuscript. All authors contributed substantially to the of content.

## Conflict of Interest

The authors declare that the research was conducted in the absence of any commercial or financial relationships that could be construed as a potential conflict of interest.

## Publisher’s Note

All claims expressed in this article are solely those of the authors and do not necessarily represent those of their affiliated organizations, or those of the publisher, the editors and the reviewers. Any product that may be evaluated in this article, or claim that may be made by its manufacturer, is not guaranteed or endorsed by the publisher.
